# Hereditary Hypopituitarism

**Published:** 1914-01

**Authors:** 


Hereditary Hypopituitarism. Miss Nellie Lambert of London, is said to weigh 563 pounds, and is alleged to be the heaviest woman in the world. She is a great-granddaughter of Daniel Lambert, of Leicester, England, also reputed to have been the heaviest man of his time. This would seem to be an instance of hereditary hypopituitarism, a condition which, however, if associated with genital infantilism, should ultimately prove self-exterminative. —*Boston Med. Jour.*

